# Respiratory Virus Coinfection Is a Risk Factor for Adverse Outcomes During *Staphylococcus aureus* Bacteremia

**DOI:** 10.1093/ofid/ofag113

**Published:** 2026-03-03

**Authors:** Katherine Roberts, Simon Dewar, Rebecca K Sutherland, Clark D Russell

**Affiliations:** Clinical Infection Research Group, Western General Hospital, Edinburgh, UK; Clinical Infection Research Group, Western General Hospital, Edinburgh, UK; Clinical Infection Research Group, Western General Hospital, Edinburgh, UK; Clinical Infection Research Group, Western General Hospital, Edinburgh, UK; The University of Edinburgh Centre for Inflammation Research, Institute for Regeneration and Repair, Edinburgh, UK

**Keywords:** bacteremic *S. aureus* pneumonia, respiratory virus coinfection

## Abstract

**Background:**

We aimed to determine the impact of respiratory virus coinfection on clinical characteristics and outcomes of *Staphylococcus aureus* bacteremia (SAB).

**Methods:**

We conducted an analysis within a retrospective observational cohort study of consecutive adults with monomicrobial SAB between 08/01/2021 and 29/12/2024 in Southeast Scotland. Variables were compared between patients tested/not tested for respiratory viruses, then between patients with/without coinfection detected. Survival was compared using Kaplan-Meier curves. Multiple logistic regression was used to identify independent risk factors for mortality.

**Results:**

We identified 651 patients with SAB during the study period; 64.5% (420/651) underwent polymerase chain reaction testing for respiratory viruses, 9.1% of whom (38/420) tested positive (severe acute respiratory syndrome coronavirus 2, n = 30; influenza A, n = 7; respiratory syncytial virus, n = 1). There were no differences in baseline characteristics between those testing positive vs negative for respiratory virus coinfection, including age, sex, Charlson Comorbidity Index, and quick Sequential Organ Failure Assessment score. Presence of coinfection was associated with a respiratory portal of entry of SAB, that is, bacteremic pneumonia (21.1% with coinfection vs 5.2% without coinfection; *P* = .002). Patients with respiratory virus coinfection had higher 30-day all-cause mortality (31.6% vs 18.0%; *P* = .04). Logistic regression identified that bacteremic pneumonia, but not viral coinfection itself, was independently associated with mortality. Mortality was not associated with receipt of immunomodulatory treatment of coronavirus disease 2019.

**Conclusions:**

Respiratory virus coinfection is a risk factor for bacteremic *S. aureus* pneumonia, which is associated with increased 30-day mortality, independent of age, comorbidity, and receipt of immunomodulatory treatments.

## Graphical Abstract

**Graphical Abstract:**
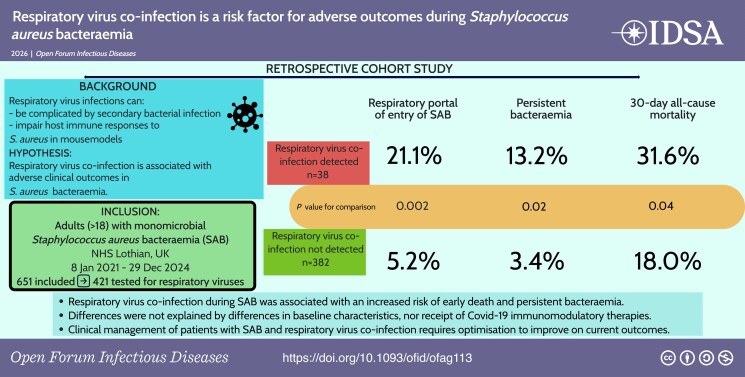
This graphical abstract is also available at Tidbit: https://tidbitapp.io/tidbits/we-aimed-to-test-the-hypothesis-that-respiratory-virus-co-infection-was-associated-with-adverse-clinical-outcomes-in-sab?utm_campaign=tidbitlinkshare&utm_source=IO

Respiratory virus infection in humans can be complicated by secondary bacterial pneumonia caused by gram-positive cocci, including *Staphylococcus aureus,* which is associated with increased mortality [1, 2, 3, 4, 5]. Mouse models of respiratory virus–*S. aureus* coinfection demonstrate that viral infection lowers the threshold for asymptomatic *S. aureus* nasal colonization to progress to pneumonia [4, 6]. Mechanistically, influenza A virus infection impairs phagocyte NADPH oxidase activity and pulmonary interleukin 17 production in mice in response to *S. aureus* [7, 8]. These factors are both key elements of the host response to *S. aureus* [9]. We therefore reasoned that respiratory virus infection could impact the clinical characteristics and outcome of *S. aureus* bacteremia (SAB) and aimed to test the hypothesis that respiratory virus coinfection was associated with adverse clinical outcomes in SAB.

## METHODS

### Study Design and Setting

We analyzed data from an ongoing retrospective cohort study of consecutive adults (aged ≥18 years) with monomicrobial SAB in the United Kingdom (Edinburgh and the Lothians), as previously described [10, 11, 12]. This study was approved by the South East Scotland Research Ethics Committee 02 (23/SS/0025/AM02). Patients were identified through a laboratory database search for all blood cultures with growth of *S. aureus* from January 8, 2021, to December 29, 2024. These dates were chosen to avoid inclusion of large numbers of people with COVID-19 before the availability of vaccination and therapeutics, who were thus unrepresentative of the current clinical characteristics of coronavirus disease 2019 (COVID-19).

### Respiratory Virus Testing

Results of respiratory virus testing performed between 7 days before and 3 days after the index positive blood culture were included. Testing was performed at the discretion of clinical teams and targeted either severe acute respiratory syndrome coronavirus 2 (SARS-CoV-2) alone or SARS-CoV-2, influenza A, influenza B, and respiratory syncytial virus (RSV). Respiratory virus testing was done using either point-of-care or routine laboratory-based testing. The point-of-care analyzers used were Cepheid GeneXpert, Roche Liat, and Roche Eplex. Laboratory analyzers used were Alinity M (Abbott) and Seegene (Mast).

### Definitions and Outcomes

The portal of entry of bacteremia was the most likely entry point of *S. aureus* into the bloodstream, determined retrospectively based on clinical findings, microbiology results, radiology, and documentation from Infectious Diseases consults. Metastatic infection was defined as the identification of foci of infection remote from the portal of entry, arising through hematogenous dissemination. Assignment of patients to clinical subphenotypes was taken from previous analyses of this cohort [11, 12]. All-cause mortality was recorded 30 days after the index positive blood culture. Persistent SAB was defined as a positive blood culture for *S. aureus* >48 hours after the index blood culture while still receiving treatment.

### Analysis

Variables were compared between patients tested/not tested for respiratory viruses, then between patients with/without coinfection detected. Categorical variables were compared using the Fisher exact or chi-square test, and continuous variables were compared using the Mann-Whitney *U* or Kruskal-Wallis test with correction for multiple testing where appropriate. Survival was compared using Kaplan-Meier curves and the log-rank test. Multiple logistic regression was used to identify independent risk factors for mortality. R (version 4.2.2) and GraphPad Prism (version 10.5.0 for macOS) were used for data analysis.

## RESULTS

We identified 651 patients with SAB during the study period (Supplementary Figure 1); 64.5% (n = 420/651) underwent respiratory virus testing for either SARS-CoV-2 alone (n = 101) or SARS-CoV-2, influenza A, influenza B, and RSV (n = 319). Thirty-eight of these 420 tested patients (9.1%) tested positive for 1 respiratory virus. Thirty tested positive for SARS-CoV-2, 7 tested positive for influenza A, and 1 tested positive for RSV.

### Clinical Characteristics

There were no differences in most baseline characteristics between patients tested for respiratory viruses and those not tested, including age, sex, Charlson Comorbidity Index (CCI), and quick Sequential Organ Failure Assessment (qSOFA) score (Supplementary Table 1). Consistent with enhanced infection prevention and control practices in hemodialysis units, patients undergoing hemodialysis were more likely to be tested. People who inject drugs were less likely to be tested. The portal of entry of bacteremia also differed between tested/not tested patients, with an intravenous catheter and the respiratory tract both more common among patients undergoing testing.

**Figure 1.**
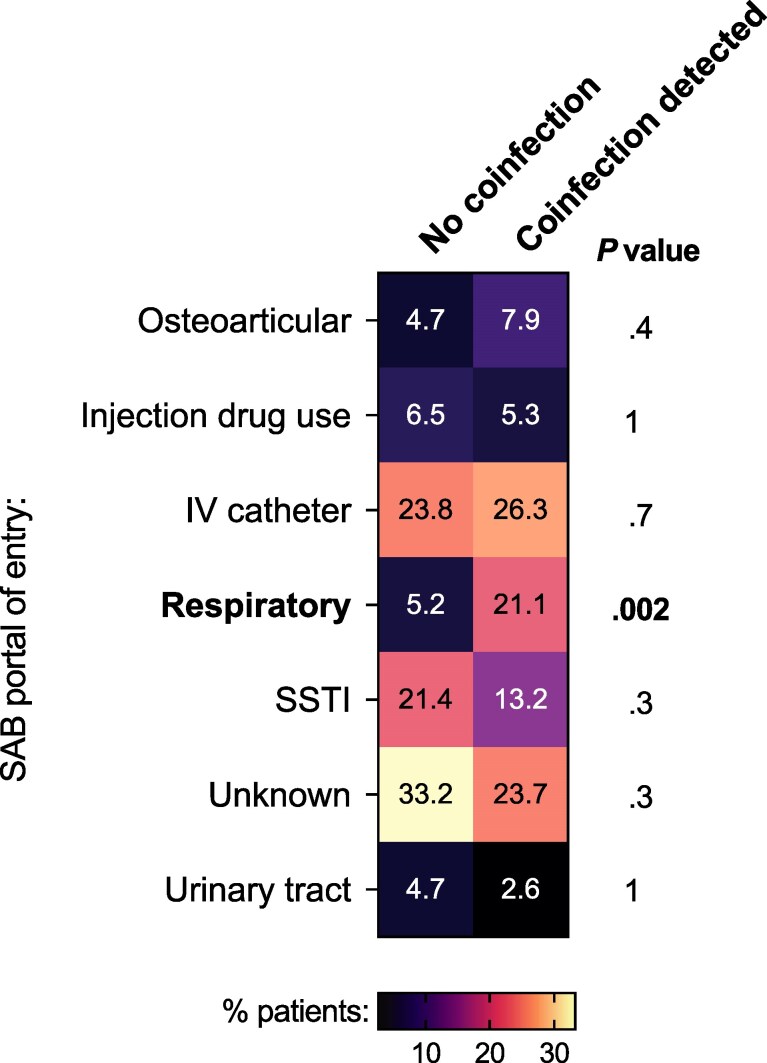
Relationship between portal of entry and respiratory virus coinfection during SAB. Heatmap shaded by percentage of patients within each group. Numbers inside cells are the percentages of patients in coinfection groups with the specified portal of entry of SAB. Each portal of entry was compared between groups using the Fisher exact test. *P* values shown are unadjusted; to adjust for multiple comparisons, the significance level was set at .007 (α = .05; n = 7). Abbreviations: IV, intravenous; SAB, *Staphylococcus aureus* bacteremia; SSTI, skin or soft tissue infection.

Within the tested group (n = 420), baseline characteristics also did not differ significantly between patients with/without a coinfection detected, including age, sex, CCI, and qSOFA score (Supplementary Table 2). Patients with coinfection were substantially more likely to have SAB originating from the respiratory tract, that is, bacteremic pneumonia (21.1% vs 5.2%; *P* = .002) (Figure 1). There was a nonsignificant trend toward an association between coinfection and health care–related acquisition (Supplementary Table 2). There were no differences in infection with MRSA or isolates encoding the Panton-Valentine leucocidin (Supplementary Table 2).

We have previously described 5 clinically distinct and reproducible subphenotypes of SAB [11, 12]. When we stratified results of respiratory virus testing by subphenotype membership, we identified higher rates of testing in subphenotype D (SAB associated with chronic kidney disease; 78.4% tested) and lower rates in subphenotype E (SAB associated with injection drug use; 46.2% tested) but no statistically significant difference in detection of coinfection between subphenotypes (Supplementary Table 3).

### Outcomes

**Figure 2.**
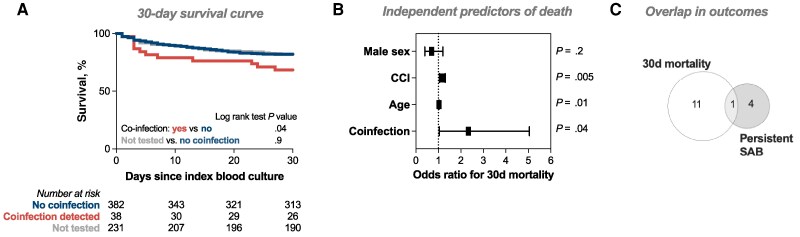
Outcomes of respiratory virus coinfection during SAB. A, Kaplan-Meier survival curve for unadjusted 30-day survival. Groups compared using the log-rank test. B, Results of multiple logistic regression with 30-day mortality as the outcome. Graph shows odds ratio and 95% CI. C, Euler diagram representing overlap in number of patients who died within 30 days of index blood culture and who were diagnosed with persistent SAB. Abbreviations: CCI, Charlson Comorbidity Index; SAB, *Staphylococcus aureus* bacteremia.

There was no significant difference in the incidence of metastatic complications between patients with/without coinfection, but patients with coinfection were more likely to have persistent bacteremia (13.2% vs 3.4%; *P* = .02). Thirty-day all-cause mortality was also higher among patients with coinfection (31.6% vs 18.0%; *P* = .04), with the difference mainly driven by deaths within the first 10 days since index blood culture (Figure 2A). Respiratory virus coinfection remained independently associated with mortality after adjusting for age, sex, and comorbidity (odds ratio, 2.3; 95% CI, 1.03–5.05; *P* = .04) (Figure 2B). Thirty-day mortality and persistent bacteremia represented largely distinct outcomes, but this could reflect the competing risk of death when assessing persistent bacteremia (Figure 2C).

### Bacteremic Pneumonia

**Figure 3.**
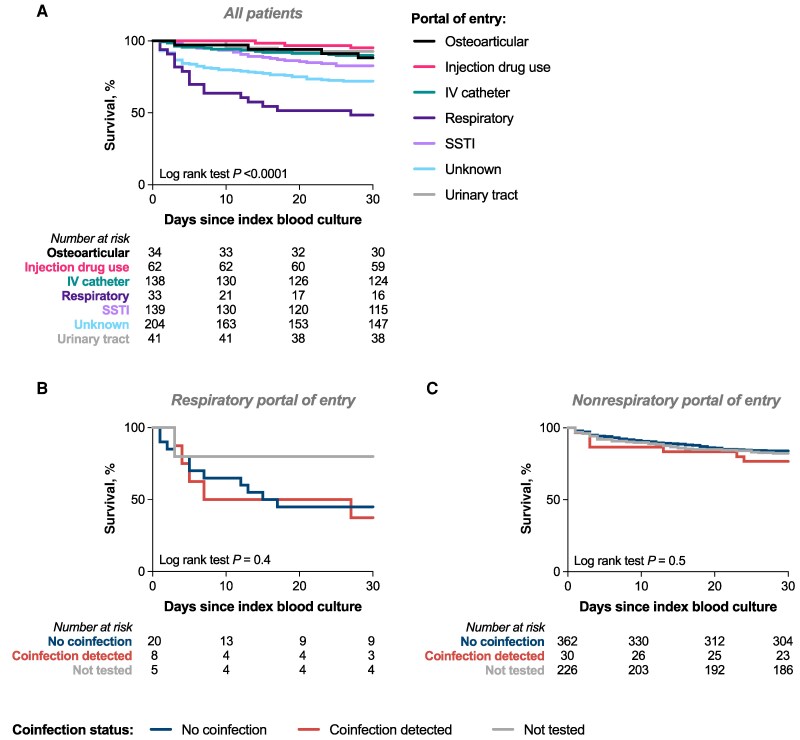
Relationship between portal of entry, respiratory virus coinfection, and survival. Kaplan-Meier survival curves showing unadjusted 30-day survival for (A) all patients, stratified by portal of entry, (B) patients with SAB from respiratory portal of entry, stratified by respiratory virus coinfection status, and (C) patients with SAB from all other (nonrespiratory) portals of entry, stratified by respiratory virus coinfection status. Groups were compared using the log-rank test. Abbreviations: IV, intravenous; SAB, *Staphylococcus aureus* bacteremia; SSTI, skin or soft tissue infection.

Among all patients, a respiratory portal of entry for SAB (bacteremic *S. aureus* pneumonia) had the highest 30-day all-cause mortality (Figure 3A). When bacteremic *S. aureus* pneumonia was considered separately, there was no increase in mortality associated with detection of respiratory virus coinfection compared with no coinfection (Figure 3B). Similarly, no difference was observed when the survival analysis was restricted to a nonrespiratory portal of entry (Figure 3C). Finally, when portal of entry was included in the multiple logistic regression analysis, respiratory virus coinfection was no longer independently associated with mortality (odds ratio [OR], 1.5; 95% CI, 0.6–3.6; *P* = .3), whereas respiratory portal of entry was (OR, 10.3; 95% CI, 4.1–27.0; *P* < .0001). We therefore conclude that the increase in mortality associated with respiratory virus coinfection is mediated by the respiratory origin of these bacteremias. No instances of persistent bacteremia occurred in people with respiratory tract origin of SAB.

### Inflammatory Markers

**Figure 4.**
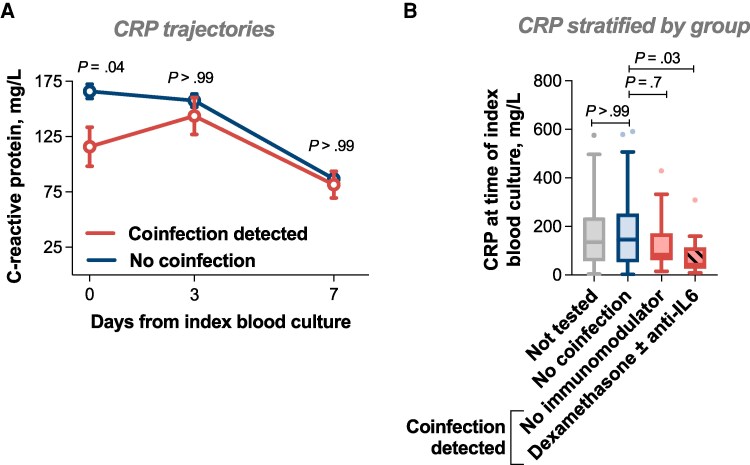
Relationship between respiratory virus coinfection and COVID-19 immunomodulatory therapies and C-reactive protein. A, Line graph representing CRP concentration on days 0, 3, and 7 from index blood culture. Graph shows mean and standard error of the mean for each time point. Groups compared using the Kruskal-Wallis test with Dunn's test for multiple comparisons. B, Box and whisker plot drawn using Tukey's method. Box shows interquartile range, and horizontal line shows median. Groups were compared using the Kruskal-Wallis test with Dunn's test for multiple comparisons. Abbreviations: COVID-19, coronavirus disease 2019; CRP, C-reactive protein.

C-reactive protein (CRP) concentration at the time of index blood culture (day 0) was lower in patients with coinfection, then increased to a similar concentration by day 3, appearing to represent a lag (Figure 4A). There were no differences in the day 0 leucocyte or platelet counts associated with detection of coinfection (Supplementary Figure 2).

### Impact of COVID-19 Immunomodulatory Therapies

Of 30 patients with SARS-CoV-2 coinfection, 11 received dexamethasone alone, and 1 received dexamethasone plus tocilizumab. The lowest median day 0 CRP was seen in patients receiving these COVID-19 immunomodulatory treatments, though there was still a trend toward lower CRP in the patients with coinfection not receiving these treatments (Figure 4B). Within the limits of the small data set, receipt of immunomodulatory therapy did not appear to increase the likelihood of 30-day mortality or persistent SAB (Supplementary Table 4).

## DISCUSSION

Respiratory virus coinfection during SAB was associated with an increased risk of early death and persistent bacteremia. These differences were not explained by differences in baseline characteristics, such as age, sex, or comorbidity; nor receipt of COVID-19 immunomodulatory therapies. The association with increased mortality appears to be mediated by an increased risk of bacteremic *S. aureus* pneumonia in people with coinfection. Interestingly, among patients with bacteremic *S. aureus* pneumonia, detection of respiratory virus coinfection was not associated with increased mortality.

Our study has some important limitations. The findings are from a single center and require validation in additional cohorts to ensure generalizability. Only 64.5% of patients underwent testing for respiratory virus coinfection, and this was based on clinical suspicion, not systematic testing, meaning that there will be a selection bias. The sample size of patients who tested positive was small (n = 38), reducing statistical power, and the findings predominantly relate to SARS-CoV-2 coinfection. There is a competing risk between mortality and persistent bacteremia, meaning that patients who died early may not have had the opportunity to be diagnosed with persistent SAB, potentially underestimating the association between coinfection and persistent bacteremia. Time to receipt of antimicrobials active against *S. aureus* was not recorded, and it is possible that delayed receipt of antimicrobials in patients with confirmed/suspected respiratory virus infection could also explain the worse outcomes in this group. Finally, predisposing factors in patients with respiratory origin SAB without respiratory virus coinfection were not documented.

Bacteremic *S. aureus* pneumonia is recognized as a relatively uncommon entity but is reproducibly associated with high mortality relative to SAB originating from alternative portals of entry [13, 14, 15]. Previously described risk factors include intensive care unit admission and intravenous drug use [16]. Our study adds respiratory virus coinfection as a risk factor. Based on observations from mouse models [4, 6, 7, 8], we hypothesize that the adverse outcomes in the coinfected group relate to virus-mediated interference with host defense in the lower respiratory tract, resulting in systemic invasion of *S. aureus* after development of pneumonia. The impaired antibacterial responses underlying this could represent treatable traits and be generalizable to other contexts where *S. aureus* enters the bloodstream from the respiratory tract, for example, ventilator-associated pneumonia in critical illness. Neutrophil dysfunction occurs in adults with critical illness, and this defective ex vivo neutrophil phagocytosis can be reversed with administration of granulocyte-macrophage colony-stimulating factor to critically ill patients [17]. Ex vivo administration of interferon gamma to neutrophils from critically ill adults can reverse defective phagocytosis and improve effector functions, including reactive oxygen species production and killing of *S. aureus* [18]. Interferon gamma administration has also been shown to improve monocyte dysfunction in adults with septic shock [19]. Future work should investigate host responses during *S. aureus*–respiratory virus coinfection in humans to determine if the observed deficits (eg, NADPH oxidase, interleukin-17) described in mice also apply to humans and could therefore represent targets for host-directed therapies. In conclusion, clinical management of patients with SAB and respiratory virus coinfection requires optimization to improve on current outcomes. Such strategies could have broader utility in the setting of secondary gram-positive bacterial pneumonia following respiratory virus infection.

## Supplementary Material

ofag113_Supplementary_Data

## Supplementary Data

Supplementary materials are available at *Open Forum Infectious Diseases* online. Consisting of data provided by the authors to benefit the reader, the posted materials are not copyedited and are the sole responsibility of the authors, so questions or comments should be addressed to the corresponding author.
