# Supplementary material for: Respiratory Virus Coinfection Is a Risk Factor for Adverse Outcomes During Staphylococcus aureus Bacteremia
Source: Open Forum Infect Dis. 2026 Mar 3;13(3):ofag113. doi: 10.1093/ofid/ofag113 (PMC12996906; doi:10.1093/ofid/ofag113)
Supplement: ofag113_Supplementary_Data [file ofag113_supplementary_data.pdf]

Respiratory virus co-infection is a risk factor for adverse outcomes during *Staphylococcus aureus* bacteraemia

Supplementary material

For data sharing, additional information, or collaborations, please contact Clark Russell, University of Edinburgh (clark.russell@ed.ac.uk)

Contents:

Supplementary Table 1: Baseline characteristics, tested vs. not tested for respiratory viruses 2

Supplementary Table 2: Baseline characteristics, co-infected detected vs. not detected 3

Supplementary Table 3: Results stratified by clinical subphenotype 4

Supplementary Table 4: Impact of Covid-19 immunomodulatory therapies 5

Supplementary Figure 1: Flow diagram for Edinburgh retrospective observational cohort 6

Supplementary Figure 2: Peripheral blood cell counts at time of index blood culture 7

References 8

Supplementary Table 1: Baseline characteristics, tested vs. not tested for respiratory viruses

	Tested<br>Median (IQR) / Percentage (n/420)	Not tested<br>Median (IQR) / Percentage (n/231)	p-value
**Age (years)**	67 (53, 78)	65 (48, 80)	0.7
**Male sex**	63.6% (267)	69.7% (161)	0.1
**qSOFA score**	1 (0, 1)	1 (0, 1)	0.2
**Person who injects drugs**	7.4% (31)	15.6% (26)	0.002
**Surgery in 30d prior to index blood culture**	8.1% (34)	9.1% (21)	0.7
**Comorbidities**			
Charlson Comorbidity Index	4 (2, 7)	4 (2, 7)	0.2
Diabetes Mellitus (all)	26.9% (113)	29.4% (68)	0.9
Heart Failure	19% (80)	18.6% (43)	0.9
Stroke	13.6% (57)	17.3% (40)	0.2
Diabetes Mellitus with complications	12.9% (54)	13% (30)	1
Myocardial Infarction	11.7% (49)	12.6% (29)	0.8
Chronic Obstructive Pulmonary Disease	11.9% (50)	7.8% (18)	0.1
Localised Cancer	8.8% (37)	9.5% (22)	0.8
Dementia	6.2% (26)	10.4% (24)	0.07
Chronic Kidney Disease	9% (38)	4.3% (10)	0.03
Connective Tissue Disease	9.3% (39)	9.5% (22)	1
Metastatic Cancer	7.1% (30)	5.2% (12)	0.4
Peripheral Vascular Disease	6.7% (28)	4.3% (10)	0.3
Moderate-Severe Liver Disease	6.2% (26)	5.6% (13)	0.9
Haemodialysis	4.8% (20)	0.9% (2)	0.006
**Site of acquisition**			
Community-acquired non-healthcare-associated	40% (168)	42.9% (99)	
Community-acquired healthcare-associated	28.3% (119)	22.9% (53)	
Nosocomial	35.7% (150)	34.2% (79)	0.4
**Time from hospital admission to first positive blood culture (days)**	0 (0, 4)	1 (0, 5)	0.5
**Portal of entry**			
Unknown	32.4% (136)	27.3% (63)	0.2
Intravenous catheter (of any kind)	24% (101)	16% (37)	0.02
Skin or soft tissue infection	20.7% (87)	22.5% (52)	0.6
Injection drug use	6.4% (27)	15.2% (35)	0.0004
Urinary	4.5% (19)	9.5% (22)	0.02
Bone	5% (21)	5.6% (13)	0.7
Respiratory	6.7% (28)	2.2% (5)	0.01
**Metastatic foci present**	29.5% (124)	26.4% (61)	0.4
**Microbiology**			
Methicillin-resistent S. aureus	1.9% (8)	5.2% (12)	0.03
PVL toxin genes detected	0.7% (3)	2.2% (5)	0.1

Categorical variables were compared using Fisher's Exact or Chi-squared tests and continuous variables were compared using the Mann Whitney U test. Missing data were removed for qSOFA score (47 missing for participants not tested and 71 missing for tested) and for PVL toxin genes detected (4 missing for not tested participants and 8 missing for tested).

Supplementary Table 2: Baseline characteristics, co-infected detected vs. not detected

	Tested positive Median (IQR) / Percentage (n/38)	Tested negative Median (IQR) / Percentage (n/382)	p-value
**Age (years)**	69 (57.25, 79)	67 (52, 78)	0.3
**Male sex**	60.5% (23)	63.9% (244)	0.7
**qSOFA score**	1 (0, 2)	1 (0, 1)	0.8
**Person who injects drugs**	5.3% (2)	7.6% (29)	1
**Surgery in 30d prior to index blood culture**	5.3% (2)	8.4% (32)	0.8
**Comorbidities**			
Charlson Comorbidity Index	4 (2.75, 5.25)	4 (2, 7)	0.4
Diabetes Mellitus (all)	21.1% (8)	27.5% (105)	0.4
Heart Failure	13.1% (5)	19.6% (75)	0.4
Stroke	2.6% (1)	14.7% (56)	0.04
Diabetes Mellitus with complications	13.1% (5)	12.8% (49)	1
Myocardial Infarction	21.1% (8)	10.7% (41)	0.07
Chronic Obstructive Pulmonary Disease	13.2% (5)	11.7% (45)	0.8
Localised Cancer	2.6% (1)	9.4% (36)	0.2
Dementia	5.3% (2)	6.3% (24)	1
Chronic Kidney Disease	2.6% (1)	9.7% (37)	0.2
Connective Tissue Disease	18.4% (7)	8.4% (32)	0.07
Metastatic Cancer	5.3% (2)	7.3% (28)	1
Peripheral Vascular Disease	5.3% (2)	6.8% (26)	1
Moderate-Severe Liver Disease	5.3% (2)	6.3% (24)	1
Haemodialysis	0% (0)	5.2% (20)	0.2
**Site of acquisition**			
Community-acquired non-healthcare-associated	23.7% (9)	41.6% (159)	
Community-acquired healthcare-associated	36.8% (14)	25.4% (97)	
Nosocomial	39.5% (15)	33% (126)	0.09
**Time from hospital admission to first positive blood culture (days)**	0.5 (0, 8.5)	0 (0, 4)	0.5
**Portal of entry**			
Unknown	23.7% (9)	33.2% (127)	0.3
Intravenous catheter (of any kind)	26.3% (10)	23.8% (91)	0.7
Skin or soft tissue infection	13.2% (5)	21.4% (82)	0.3
Injection drug use	5.3% (2)	6.5% (25)	1
Urinary	2.6% (1)	4.7% (18)	1
Bone	7.9% (3)	4.7% (18)	0.4
Respiratory	21.1% (8)	5.2% (20)	0.002
**Metastatic foci present**	23.7% (9)	30.1% (115)	0.5
**Microbiology**			
Methicillin-resistent S. aureus	2.6% (1)	1.8% (7)	0.5
PVL toxin genes detected	2.6% (1)	0.5% (2)	0.2

Categorical variables were compared using Fisher's Exact or Chi-squared tests and continuous variables were compared using the Mann Whitney U test. Missing data were removed for qSOFA score (5 missing for positive participants and 47 missing for negative participants) and for PVL toxin genes detected (2 missing for positive participants and 6 missing for negative participants).

Supplementary Table 3: Results stratified by clinical subphenotype

	Clinical subphenotype					*P*-value
	**A**	**B**	**C**	**D**	**E**	
Number in subphenotype	188	195	152	51	65	NA
Respiratory virus testing performed, n(%)	114 (60.6)	130 (66.7)	106 (69.7)	40 (78.4)	30 (46.2)	0.002
Co-infection detected[a], n(%)	9 (7.9)	18 (13.8)	8 (7.5)	1 (2.5)	2 (6.7)	0.2

Categorical variables were compared using Fisher's Exact or Chi-squared tests.

[a]Using the number of tested patients as denominator.

Supplementary Table 4: Impact of Covid-19 immunomodulatory therapies

	**Dexamethasone ± tocilizumab**	**No therapy**
30-day mortality	4/12	4/18
Persistent bacteraemia	0/12	5/18

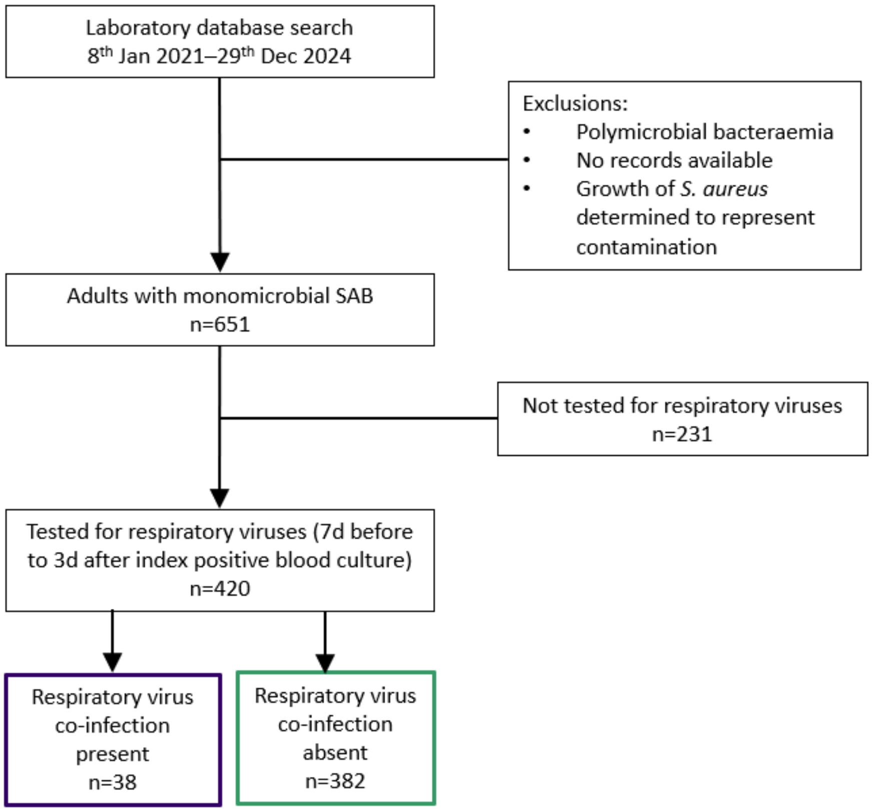

Supplementary Figure 1: Flow diagram for Edinburgh retrospective observational cohort

Testing: either SARS-CoV-2 alone (n=101), or SARS-CoV-2, influenza A, influenza B, and respiratory syncytial virus (RSV) (n=319). Previous analyses using this cohort have been published[1-3]

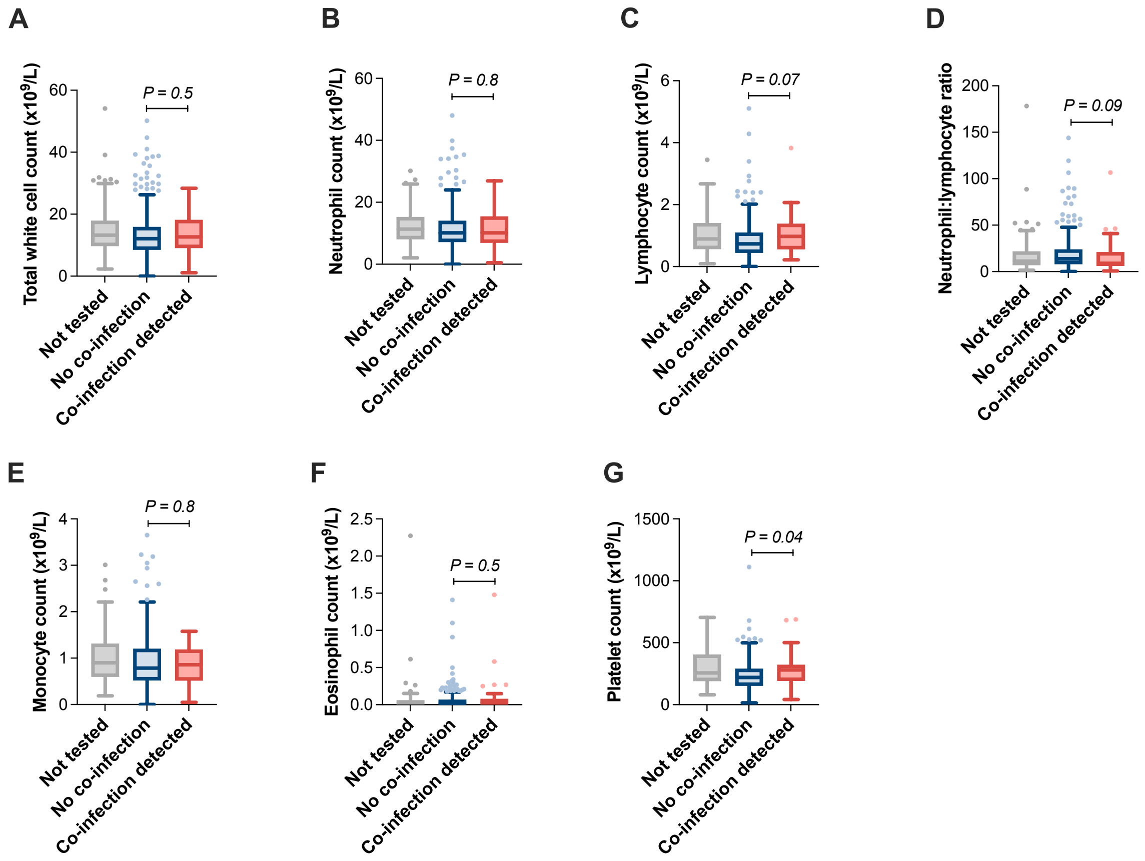

Supplementary Figure 2: Peripheral blood cell counts at time of index blood culture

(A) total white cell count, **(B)** neutrophil count, **(C)** lymphocyte count, **(D)** neutrophil:lymphocyte ratio, **(E)** monocyte count, **(F)** eosinophil count, **(G)** platelet count. Box and whisker plots drawn using Tukey's method. Boxes show interquartile range and horizontal lines show median. 'No co-infection' group compared with 'co-infection detected' group using Mann-Whitney tests. P-values shown are unadjusted; to adjust for multiple comparisons the significance level was set at 0.007 (α=0.05, n=7).

References

1. Russell CD, Berry K, Cooper G, et al. Distinct Clinical Endpoints of Staphylococcus aureus Bacteraemia Complicate Assessment of Outcome. Clin Infect Dis **2024**; 79(3): 604-11.
2. Swets MC, Bakk Z, Westgeest AC, et al. Clinical Subphenotypes of Staphylococcus aureus Bacteremia. Clin Infect Dis **2024**; 79(5): 1153-61.
3. Swets MC, Bakk Z, Westgeest AC, et al. Reproducible identification of Staphylococcus aureus bacteremia clinical subphenotypes. Clin Infect Dis **2025**.
